# Subjective Symptoms in Magnetic Resonance Imaging Personnel: A Multi-Center Study in Italy

**DOI:** 10.3389/fpubh.2021.699675

**Published:** 2021-10-07

**Authors:** Giulia Bravo, Alberto Modenese, Giulio Arcangeli, Chiara Bertoldi, Vincenzo Camisa, Gianluca Corona, Senio Giglioli, Guido Ligabue, Roberto Moccaldi, Nicola Mucci, Martina Muscatello, Irene Venturelli, Luigi Vimercati, Salvatore Zaffina, Giulio Zanotti, Fabriziomaria Gobba

**Affiliations:** ^1^Department of Biomedical, Metabolic and Neural Sciences, University of Modena and Reggio Emilia, Modena, Italy; ^2^Department of Medicine, University of Udine, Udine, Italy; ^3^Department of Experimental and Clinical Medicine, University of Florence, Florence, Italy; ^4^Occupational Medicine Unit, Bambino Gesù Children's Hospital—IRCCS, Rome, Italy; ^5^Health Surveillance Service, University Hospital Policlinico, Modena, Italy; ^6^Occupational Medicine Unit, Azienda Usl Toscana Sud-Est, Siena, Italy; ^7^Chair of Radiology, University of Modena and Reggio Emilia, Modena, Italy; ^8^Prevention and Protection Service, National Research Council, Rome, Italy; ^9^Interdisciplinary Department of Medicine, Occupational Medicine “B. Ramazzini” Unit, University of Bari, Bari, Italy

**Keywords:** magnetic resonance imaging, electromagnetic fields exposure, subjective symptoms, MRI operators, occupational health

## Abstract

**Introduction:** Magnetic Resonance Imaging (MRI) personnel have significant exposure to static and low-frequency time-varying magnetic fields. In these workers an increased prevalence of different subjective symptoms has been observed. The aim of our study was to investigate the prevalence of non-specific subjective symptoms and of “core symptoms” in a group of MRI personnel working in different centers in Italy, and of possible relationships with personal and occupational characteristics.

**Methods:** The occurrence of 11 subjective symptoms was evaluated using a specific questionnaire with 240 subjects working in 6 different Italian hospitals and research centers, 177 MRI health care and research personnel and 63 unexposed subjects employed in the same departments. Exposure was subjectively investigated according to the type of MRI scanner (≤1.5 vs. ≥3 T) and to the number of MRI procedures attended and/or performed by the personnel, even if no information on how frequently the personnel entered the scanner room was collected. The possible associations among symptoms and estimated EMF exposure, the main characteristics of the population, and job stress perception were analyzed.

**Results:** Eighty-six percent of the personnel reported at least one symptom; drowsiness, headache, and sleep disorders were the most frequent. The total number of symptoms did not differ between exposed persons and controls. Considering the total number of annual MRI procedures reported by the personnel, no significant associations were found nor with the total number of symptoms, nor with “core symptoms.” Only subjects complaining of drowsiness also reported a significantly higher mean annual number of MRI procedures with ≤ 1.5 T scanners when compared with exposed subjects without drowsiness. In a multivariate model, subjects with a high level of perceived stress complained of more symptoms (*p* = 0.0002).

**Conclusions:** Our study did not show any association between the occurrence of reversible subjective symptoms, including the more specific “core symptoms,” and the occupational exposure of MRI personnel to static and low-frequency time-varying magnetic fields. On the other hand, the role played by occupational stress appears to be not negligible. In further research in this field, measurements of EMF exposure should be considered.

## Introduction

The number of Magnetic Resonance Imaging (MRI) scanners, and consequently the number of patients involved in MRI procedures and the personnel exposed to MRI-emitted electromagnetic fields (EMFs) have constantly increased since the early ‘90s in Europe and worldwide (1). The strength of the static magnetic flux density of the scanners currently used for diagnostic purposes is most commonly between 1 and 3 Tesla, though MRI scanners used for both clinical and research purposes can reach higher strengths (2, 3).

Considering occupational exposure, MRI personnel are potentially exposed to significant levels of static magnetic fields (SMFs), which are continuously active even when the scanner is not acquiring images, as they are associated with a powerful magnet. Moreover, workers within the SMF are also exposed to time-varying electric and magnetic fields when they perform movements close to the magnet. Contrary to patients, MRI personnel are usually not exposed to the radio-frequency (RF) EMFs generated by the scanner during the acquisition of diagnostic images, with a few possible exceptions when the personnel need to stay very close to patients and to the bore, as may sometimes happen, for example, in the case of pediatric or non-autonomous patients (4–7).

The risk related to occupational exposure to these types of EMFs needs to be evaluated, as it is for other occupational risks, in order to ensure adequate occupational safety and health (OSH) of MRI personnel (8). Among the effects to be considered for appropriate preventive interventions, it should be noted that, according to the scientific literature, various subjective sensory symptoms have been reported for MRI personnel (1, 9–15). These effects can be related to the mechanisms of current induction in electro-sensitive tissues of the human body by the SMFs and by the low frequency magnetic fields (4–6). Even though the majority of the reported symptoms are non-specific, a group of five “core symptoms” have been suggested: vertigo, nausea, head ringing, magnetophosphenes, and metallic taste (12). On the other hand, a number of more general symptoms have often been reported, such as headache and concentration problems, unusual drowsiness or tiredness, balance problems, and others (1, 9–15). Even though all these effects seem rapidly reversible when EMF exposure of MRI personnel is interrupted, their prevention is relevant because of the possibility of a higher risk of accidents leading to injury, especially after work-shift exposure (16, 17). There are also various experimental observations of minor and reversible alterations in performance, balance, and cognitive tests in groups of volunteers (9, 10, 18–23), also confirmed by a recent case report of two patients falling after an MRI examination (24).

According to these premises, the aim of this study is to report, for the first time in Italy, the results of a multi-center investigation of reported non-specific subjective symptoms in a group of MRI personnel, evaluating the presence of the suggested “core symptoms” and their distribution according to the characteristics of the exposed subjects and possible confounding factors.

## Materials and Methods

### Study Setting and Population

Between June 2013 and September 2016, we performed a multi-center observational study involving personnel employed in the radiology units of six different centers in Italy: the hospitals of the cities of Modena, in northern Italy, Florence and Siena in central Italy, Bari and Rome in the south, and, also in Rome, a radiology research center of the National Research Council (CNR).

Based on some preliminary results and on the scientific literature on the subjective symptoms reported by MRI personnel (9–14), we developed an original Italian questionnaire that was previously applied during two single-center pilot studies involving a sub-sample of 17 young resident physicians attending a post-graduate medical school in radiology and engaged in MRI for <1 year (1, 15). For more details on the questionnaire, we have enclosed as Supplementary Material a full translated version of the tool used. Briefly, the questionnaire surveys 36 items, including sociodemographic and occupational characteristics, with details on the type of MRI scanners and the amount of time employed as MRI personnel, and information on the personnel's health status, related to both previous relevant diseases and drug intake, and investigating in particular 11 specific symptoms: vertigo, nausea, tinnitus, metallic taste, magnetophosphenes, headache, drowsiness, concentration problems, balance instability, memory loss, and sleep disorders. The frequency of the symptoms was estimated based on a Likert scale (never or less than once a month, at least once a month, one-four times per week, more than four times per week). The MRI personnel were also asked to indicate whether they felt that the origin of the reported symptoms was related to the MRI exposure, whether the symptoms were present during the MRI work shift, and when the symptoms appeared and disappeared in relation to the MRI shifts.

As work-related stress can be a relevant confounder for the appearance of several of the subjective symptoms investigated, a specific item of the questionnaire (item 17) was used to evaluate job stress, following the verification of internal consistency with other items investigating specific factors related to job content and context that may pose a risk of work-related stress (items 5–9). The question used provides a definition of work-related stress and asks the participants to judge whether they had experienced that kind of stress during the last 12 months on a five-point Likert-like scale (not at all, slightly, moderately, definitely, extremely). For the analysis presented in this manuscript, the five-point score was grouped into three categories: (1). Absence of job stress, corresponding to the “not at all” answer to item 17; (2). medium job stress experienced in the last year, corresponding to the answers “slightly” and “moderately”; and (3). high job stress, attributed to the answers “definitely” and “extremely.” Another important question, used mainly to verify whether there was a difference in self-perception of health conditions comparing exposed and unexposed subjects, was item 16. This question asks the participants to judge their level of agreement or disagreement on a five-point Likert scale with the statement “Your health conditions are good.” For the analysis presented in this report, we grouped together subjects who indicated agreement/extreme agreement in the category of “good health status,” comparing it with subjects who indicated another judgement.

All the questionnaires were collected anonymously and on a voluntarily basis, and the study was approved by the Institutional Review Board (reference number 2443/CE).

The inclusion criteria for the study were:

- Being an employee of a radiology unit of one of the six research and medical centers included in the multi-center study;
- Being between 18 and 70 years old;
- Voluntarily accepting to participate in the research, signing the informed consent.

We excluded subjects taking pharmaceutical drugs or in any case with diseases, diagnosed by a physician, that would interfere with the subjective symptoms evaluated.

### Exposure Evaluation

We considered MRI personnel as all health care workers (HCWs) who stated that they had worked shifts involving work with MRI scanners during the previous year. Other HCWs were considered as unexposed controls.

Unfortunately, no objective measurements for determining the levels of electromagnetic field exposure were available, so the exposure of the MRI personnel was estimated based on the questionnaire answers related to the type of scanner and the reported number of procedures, i.e., the number of patient examinations with the operator working inside what is known as the “controlled access area” (CAA) according to the European Directive 2013/35/EU, where SMF intensity >0.5 mT is expected (25, 26). It should be noted that working in the CAA does not necessarily mean that the operator enters the scanner room during the examination or is actually exposed to MRI-related EMFs. In the exposed group, we evaluated the strength of the MRI scanner used (i.e., <1.5, 1.5, or ≥3 T), the estimate of the average number of hours spent with direct involvement in MRI procedures during a work shift and the total number of MRI procedures per year reported, as well as the number of years spent working with MRI. We then considered eventual modifications of the reported symptoms' duration and occurrence in relation to the type of scanner used. For this purpose, we also performed a simple analysis grouping the MRI personnel into two categories: ≤ 1.5 vs. ≥ 3 T, adopting the criterion according to which a higher field strength could indicate higher SMF exposure, even if it is possible that some subjects included in the ≥ 3 T group have also reported procedures with the ≤ 1.5 T scanners. The full questionnaire and the database in the Supplementary Materials provide more details on how the information on the potential exposure of MRI personnel was collected.

### Statistical Analysis

Student's *t*-test (or non-parametric Wilcoxon-Mann-Whitney test) was used to compare the mean/median values between two groups of subjects (e.g., exposed vs. unexposed, men vs. women, exposed to MRI scanners with strengths of ≤ 1.5 vs. ≥ 3 T, etc.).

Chi-Square test (or Fisher's exact test) was used to evaluate the association between categorical variables. Pearson's (or Spearman's) correlation coefficient was used to analyze correlations between variables. Then, to evaluate the impact of the variables considered on the symptoms, multivariate regression models were built.

All the analyses were conducted using the statistical software package SAS version 9.4 for Windows. Statistical significance for all tests was set at a *p* ≤ 0.05.

## Results

The following subsections present the results of the study, including the characteristics of the study population divided into an MRI-exposed group and an unexposed group, according to age, BMI, gender, job title, health status, and job stress level (Table 1). In addition, the occurrence of the investigated subjective symptoms is shown in Table 2. Finally, the same symptoms were analyzed in relation to the reported annual number of MRI procedures by the personnel, also considering the strengths of the scanners, and the role of job stress.

**Table 1:** Overview of the study population (*n* = 240 subjects).

**Characteristics**	**Exposed**	**Unexposed**
	**(*n =* 177; 74%)**	**(*n =* 63; 26%)**
	***Mean** **±** **SD***	
Age in years	42.4 ± 9.9	42.5 ± 1 0.6
BMI	24.1 ± 3.7	24.1 ± 4.6
Gender	*n (%)*	
• Male	84 (47.5)	23 (36.5)
• Female	93 (52.5)	40 (63.5)
Job title		
• Medical doctor	80 (45.2)	21 (33.3)
• Other health and technical personnel	80 (45.2)	35 (55.6)
• Research staff	17 (9.6)	7 (11.1)
Perceived job stress^*^		
• Absent	54 (30.5)	24 (38.1)
• Medium	108 (61.0)	34 (54.0)
• High	14 (7.9)	5 (7.9)
Health status^*^		
• Good	144 (81.8)	49 (78.4)
• Not good	32 (18.2)	14 (21.6)

*Differences between the MRI exposed group and unexposed group are not statistically significant*.

* *Values missing for 1 exposed subject*.

**Table 2:** Frequency of reported symptoms occurring at least once a week given as *n* subjects (%).

**Symptom**	**Study population**	**Exposed**	**Unexposed**
	***n =* 240 (100%)**	***n =* 177 (74%)**	***n =* 63 (26%)**
Vertigo	11 (4.6)	9 (5.1)	2 (3.2)
Nausea	3 (1.3)	2 (1.1)	1 (1.6)
Concentration problems	20 (8.3)	15 (8.5)	5 (7.9)
Memory loss	11 (4.6)	6 (3.4)	5 (7.9)
Drowsiness	55 (22.9)	43 (24.3)	12 (19.1)
Headache	49 (20.4)	36 (20.3)	13 (20.6)
Metallic taste	2 (0.8)	2 (1.1)	0 (0.0)
Balance instability	13 (5.4)	10 (5.7)	3 (4.8)
Magnetophosphenes	1 (0.4)	0 (0.0)	1 (1.6)
Tinnitus	8 (3.3)	6 (3.4)	2 (3.2)
Sleep disorders	39 (16.3)	25 (14.1)	14 (22.2)

*None of the evaluated symptoms differed between exposed and unexposed subjects (p > 0.05)*.

### Study Population

The total number of responders included 283 subjects working in six different radiology units of hospitals and research centers in Italy. According to the exclusion criteria, 43 HCWs were excluded, resulting in a final sample of 240 subjects, between 26 and 65 years of age (mea*n* = 42.2 ± 10.1 SD), with a slightly higher percentage of women (55.4%).

The main occupations of the subjects were as follows:

- Medical doctors (radiologists, surgeons, anesthesiologists, ophthalmologists, cardiologists, and resident physicians of the same specializations): *n* = 101 (42.1% of the sample);
- Research staff: 24 subjects (10.0% of the sample);
- Other healthcare and MRI-related technical staff (mainly nurses and radiology technicians): *n* = 115 (47.9% of the sample).

Of the 240 subjects, 177 reported having worked with an MRI scanner in the previous 12 months. Accordingly, the exposed group represents 74% of the sample, and it includes 80 medical doctors (45.2%), 80 nurses and technicians (45.2%) and 17 researchers (9.6%). In the exposed group, 114 subjects (64.4%) worked with MRI scanners of intensity ≤ 1.5 T, with a mean number of procedures in the previous year of 682, while 63 subjects (35.6%) used also, or exclusively, scanners ≥ 3 T with an average number of procedures equal to 238. Only 10 subjects reported the use of both 3 T and ≥ 3 T scanners.

No significant differences were detected for any occupational variable or for any sociodemographic characteristic between the exposed group and the unexposed group, including gender (*p* = 0.13). We found no significant differences in job stress perception between exposed and unexposed subjects. A high level of work-related stress was perceived in a relatively small percentage of subjects: 7.9% of both the exposed and unexposed subjects (*p* = 0.547). The health status of the subjects was subjectively evaluated as “good” by 81.8% of subjects in the exposed group and by 78.4% of subjects in the unexposed group (*p* = 0.788) (Table 1).

### Evaluation of Symptoms: Characteristics and Frequency

Overall, the majority of the subjects (86.4%) complained of no less than one symptom during the last year with a frequency of at least once a month, and 42.3% of the subjects complained of symptoms with a frequency of at least once a week. No significant differences were observed between the occurrence of the symptoms in the MRI exposed group compared to the unexposed subjects.

The occurrence of subjective symptoms in the previous 12 months (with a frequency of at least once a week) is reported in Table 2.

Limiting the analysis to the five core symptoms (i.e., vertigo, nausea, head ringing, magnetophosphenes, and metallic taste), once again no significant differences in the occurrence of the symptoms were found between exposed and unexposed subjects.

Regarding the symptoms reported, in the whole population the mean number was 3.1 (±2.5 SD), with a maximum of 10 symptoms in only one subject; limiting the analysis to more frequent symptoms (at least “once a week” frequency), the mean number of reported symptoms was 0.88 (±1.4 SD).

In the whole group, no significant difference was observed between exposed and unexposed personnel considering symptoms with any frequency or with at least weekly occurrence.

Finally, regarding job characteristics, we examined the occurrence of weekly symptoms in three different occupational categories (i.e., medical doctors, nurses, and technical staff and researchers). In the whole sample, the most frequent symptoms were drowsiness, headache, and sleep disorders, reported by 22.9, 20.4, and 16.2% of subjects, respectively. No significant differences were observed for the occurrence of these symptoms in any of the three occupational groups. Even when considering only “core symptoms,” no significant differences in their occurrence were found among the three occupational groups (see Supplementary Table 1).

### Associations Between Reported Symptoms in the Exposed Group, Type of MRI Scanner Used, and Reported Number of MRI Procedures

In the exposed group, a significant increase in the mean number of symptoms was observed when comparing personnel working with scanners up to 1.5 T (mean number of symptoms = 2.8 ± 2.4 SD) with those working also, or exclusively, with scanners with a field strength ≥ 3 T (mea*n* = 3.7 ± 2.6 SD; *p* = 0.0238). However, this difference disappeared when considering only symptoms with a frequency of at least once a week (mean number in the ≥ 3 T sub-group = 0.8 ± 1.3 SD vs. 0.9 ± 1.4 SD in the ≤ 1.5 T sub-group).

Considering the total number of annual MRI procedures reported by the personnel, no significant associations were found with the total number of symptoms, nor with “core symptoms.”

As regards the specific symptoms, only subjects with drowsiness reported a mean annual number of MRI procedures with ≤ 1.5 T scanners higher when compared with exposed subjects without drowsiness (752.7 ± 802.8 SD vs. 481.0 ± 6 54.2, *p* = 0.02).

### Associations Between Subjective Symptoms in MRI Personnel, Number of MRI Procedures Reported, and Perceived Occupational Stress in a Multivariate Model

As a potential confounder, we evaluated the possible influence of job stress in relation to the occurrence of symptoms in the investigated sample. Subjects with a high level of perceived job stress complained of an average of 0.6 (±0.9 SD) core symptoms occurring at least once a week. This value was found to be lower in subjects with a medium job stress perception level (0.0 ± 0.2 SD) and in subjects without work-related stress (0.1 ± 0.5 SD). A positive association between stress levels and the average numbers of reported core symptoms per person was found (*p* = 0.0002). We then performed a multivariate regression analysis investigating the association between core symptoms, the reported number of annual MRI procedures attended and/or performed by the personnel, and scanner field strength. The model is adjusted for sex and age and includes job stress as a confounding factor. A significant association was found between stress and the reporting of core symptoms with a frequency of at least once a week, indicating a negative effect of low stress levels on symptom occurrence (OR = 0.19; CI 95%: 0.04–0.93).

## Discussion

Our study did not find significant higher reporting of subjective symptoms in the whole group of MRI personnel potentially exposed to static and low-frequency time-varying magnetic fields during MRI-related activities. We did not find an increased prevalence of symptoms when comparing exposed and unexposed subjects, or when comparing, within the sub-group of the exposed individuals, the personnel working with scanners of a field strength ≤1.5 T with those also working with ≥3 T MRI scanners, or according to the annual number of MRI procedures reported. Also considering the sub-group of “core symptoms” (12), we did not find an increased prevalence of these symptoms in the exposed subjects. This finding is partially in contrast with the results obtained by Schaap et al. suggesting an exposure-response association between exposure to SMFs and the reporting of transient symptoms, especially on the same day of the exposure and mainly considering core symptoms, and in particular vertigo (12, 13). The lack of association we found may be explained by the subjective investigation of the exposure performed, not considering, differently from these other studies, objective measurements of EMF exposure, and, moreover, not collecting information on how frequently the MRI personnel entered the scanner room, how close to the bore they were, and what kind of movements they performed. The subjective information on the exposure we collected are similar to those obtained in the study by Wilén et al. but in that case the sample was more homogeneous, including only nurses, and the authors found that the symptoms reported by the MRI personnel were related to the field strength of the magnet the nurses worked with (14).

In line with earlier research and considering these limitations of our study it should still be considered useful to evaluate the occurrence of these health complaints during health surveillance of MRI personnel (8). The importance of investigating these symptoms is not only related to their possible association with EMF exposure during MRI activities, as previously reported (12–14), but it should be considered that a systematic collection of these symptoms may help the occupational physicians responsible for the health surveillance of MRI personnel in identifying groups of subjects at “particular risk,” as indicated in European Directive 2013/35 (8). In fact, many of these symptoms, and in particular core symptoms, can be considered sensory effects of exposure to magnetic fields, and they are particularly related to the performance of physical movements within a static magnetic field. The probability of occurrence of the effects depends on the strength of the magnetic field and on the acceleration, velocity, and direction of the movement with respect to the magnetic field gradient (4–6). Furthermore, it cannot be excluded that subjects with diseases causing the same sensory effects (e.g., the Meniere's disease for vertigo) may be more susceptible to EMF effects, being therefore at a “particular risk,” even in the case of low exposure levels (8).

Another aspect to be mentioned here is the role played by perceived work stress on the investigated symptoms. The influence of work-related stress on subjective symptoms is not unexpected (1, 15), but was scarcely considered in similar studies previously conducted on MRI personnel. We found that the subjects with higher perceived stress levels reported a higher mean number of symptoms. Moreover, in the adjusted model we developed it was found that job stress levels were significantly associated with the average number of core symptoms reported by the subjects.

Our study has some limitations, some of them intrinsically related to the issue investigated. The subjective symptoms collected in this study, even though selected according to the potential relation with the action of the strong magnetic fields related to the MRI scanner during occupational activities as MRI personnel, resulted as extremely frequent in the whole group, i.e., more than 80% of the participants, regardless of exposure to MRI-related EMF fields, reported at least one of the symptoms at least once a month. The most frequent single-specific symptoms in the whole group were drowsiness, headache, and sleep disorders, involving 15–25% of the overall sample. In order to partially overcome this limitation, all the analyses in this study were done defining the group of personnel with symptomatic manifestations, based on an occurrence of the symptoms at least once a week. Moreover, it should be considered that these symptoms are also frequent in the general population, and they have various potentially relevant occupational and non-occupational factors that can play a role in their induction (27–29). For these reasons, it is important for the interpretation of the results that the composition of the studied sample was sufficiently homogeneous, as we found no significant differences in age, sex ratio, distribution according to the job categories considered, levels of perceived job stress, and perceived health status between exposed subjects, i.e., those directly involved in MRI activities within the controlled access area, and the unexposed group, working in the same department (though it should be noted that the number of unexposed workers was significantly lower compared with the number of MRI personnel). These data are relevant, as for some of the symptoms evaluated we could expect higher reporting in sub-groups of subjects such as women (e.g., for nausea, vertigo, and headache) (29, 30) or older subjects (e.g., for tinnitus, instability, sleep disorders) (31, 32). Furthermore, we excluded the subjects who reported a diagnosis of diseases and a long-term assumption of drugs that could possible interfere with the occurrence of the investigated symptoms (1, 15).

Another limitation of our research is related to the observational design of the study, according to which exposure cannot be controlled by the investigators, and issues such as the presence of bias and confounding factors are more common compared to experimental interventional studies. Moreover, in the case of this study the observation was retrospective, so that we cannot exclude a problem of recall bias in reporting the symptoms, also because the majority of the results were reported in univariate analysis showing the association between symptom occurrence and the type of MRI scanners and the number of procedures. Nevertheless, to partially overcome this limitation, we also built a multivariate regression analysis including adjustments for sex, age, and job stress levels.

Finally, a further important weakness to overcome in future studies in this field is the lack of an objective exposure evaluation. Individual exposure measurements in MRI personnel are intrinsically difficult due to several types of technical and organizational factors, but on the other hand the subjective evaluation based on job categories and on questionnaire data can give only a rough picture of the actual individual levels and can possibly be a cause of the lack of significance of the results. Furthermore, with our questionnaire mainly designed to help occupational physicians in collecting relevant clinical data useful for the health surveillance of the MRI personnel, we did not capture important information on the exposure of the subjects, e.g., how frequently they entered in the scanner rooms, the amount of time spent inside, the distance from the bore, and the actions performed, including the velocity of the movements. Moreover, the different job categories of MRI personnel considered represent a significant source of heterogeneity, as specific occupations may have specific exposures to the magnetic fields during different MRI procedures according to their roles and work tasks. Furthermore, considering the reporting of the MRI procedures by the personnel, in our analysis we did not distinguish between workers operating only with ≤ 1.5 or ≥3 T scanners, or with both, and we did not differentiate the personnel performing the procedures and those only attending. In future studies an improvement of all these abovementioned aspects is of paramount importance.

## Conclusions

Our study did not show any association between the occurrence of reversible subjective symptoms, including the more specific “core symptoms,” and the occupational exposure of MRI personnel to static and low-frequency time-varying magnetic fields. On the other hand, the role played by occupational stress appears to not be negligible. In further research in this field, measurements of EMF exposure or equivalents should be considered. To be able to perform relevant health surveillance of MRI personnel by occupational physicians it will be necessary to validate tailored questionnaires not least to identify personnel at particular risk.

## Data Availability Statement

The original contributions presented in the study are included in the article/Supplementary Material, further inquiries can be directed to the corresponding author/s.

## Ethics Statement

The studies involving human participants were reviewed and approved by Comitato Etico dell'Area Vasta Emilia Nord. The patients/participants provided their written informed consent to participate in this study.

## Author Contributions

FG and AM: conceptualization and writing—review and editing. FG, GB, and AM: methodology. GB and FG: software. GB, AM, and IV: formal analysis. GA, NM, GZ, SG, RM, VC, SZ, GC, GL, and LV: investigation. FG, GA, NM, SG, RM, VC, SZ, GC, GL, and LV: resources. GZ, IV, GB, and AM: data curation. GB, CB, MM, and IV: writing—original draft preparation. GA, FG, LV, SZ, RM, and GL: supervision. FG: project administration. All authors contributed to the article and approved the submitted version.

## Conflict of Interest

The authors declare that the research was conducted in the absence of any commercial or financial relationships that could be construed as a potential conflict of interest.

## Publisher's Note

All claims expressed in this article are solely those of the authors and do not necessarily represent those of their affiliated organizations, or those of the publisher, the editors and the reviewers. Any product that may be evaluated in this article, or claim that may be made by its manufacturer, is not guaranteed or endorsed by the publisher.
