# Supplementary material for: Subjective Symptoms in Magnetic Resonance Imaging Personnel: A Multi-Center Study in Italy
Source: Front Public Health. 2021 Oct 7;9:699675. doi: 10.3389/fpubh.2021.699675 (PMC8530375; doi:10.3389/fpubh.2021.699675)
Supplement: Supplementary file 1 [file Table_1.DOCX]

Supplementary Table. Subjective symptoms reported with a frequency of at least once a week, grouped according to the different occupational categories considered. No statistical differences have been detected.

| **Symptoms** | **Study population**  **n = 240 (100 %)** | **Medical Doctors**  **n = 101 (42.08 %)** | **Nurses/technical staff**  **n = 115 (47.92 %)** | **Researchers**  **n = 24 (10 %)** |
| --- | --- | --- | --- | --- |
| Vertigo | 11 (4.58) | 6 (2.50) | 4 (1.67) | 1 (0.42) |
| Nausea | 3 (1.25) | 2 (0.83) | 0 (0.00) | 1 (0.42) |
| Concentration problems | 20 (8.33) | 10 (4.17) | 7 (2.92) | 3 (1.25) |
| Memory loss | 11 (4.58) | 5 (2.08) | 5 (2.08) | 1 (0.42) |
| Drowsiness | 55 (22.92) | 23 (9.58) | 28 (11.67) | 4 (1.67) |
| Headache | 49 (20.42) | 20 (8.33) | 26 (10.83) | 3 (1.25) |
| Metallic taste | 2 (0.83) | 1 (0.42) | 1 (0.42) | 0 (0.00) |
| Balance instability | 13 (5.42) | 7 (2.92) | 6 (2.50) | 0 (0.00) |
| Magnetophosphenes | 1 (0.42) | 1 (0.42) | 0 (0.00) | 0 (0.00) |
| Tinnitus | 8 (3.33) | 4 (1.67) | 4 (1.67) | 0 (0.00) |
| Sleep disorders | 39 (16.25) | 14 (5.83) | 24 (10.00) | 1 (0.42) |
