# Supplementary material for: Subjective Symptoms in Magnetic Resonance Imaging Personnel: A Multi-Center Study in Italy
Source: Front Public Health. 2021 Oct 7;9:699675. doi: 10.3389/fpubh.2021.699675 (PMC8530375; doi:10.3389/fpubh.2021.699675)
Supplement: Supplementary file 3 [file Table_3.DOCX]

**SUPPLEMENTRAY MATERIAL: THE QUESTIONNAIRE**

**Personal information**

1a. Female  Male  Other 

1b. Year of birth: .............................

1. Height…………….. cm

1. Weight…………….. kg
2. Job position/title/task ……………..……………..……………..……………..

………………………………………………………………………………………………………

***Job content/context***

5. I understand how my work fits into the overall aim of the organization

| Never | Seldom | Sometimes | Often | Always |
| --- | --- | --- | --- | --- |
| 1.  | 2.  | 3.  | 4.  | 5.  |

6. I have to work very intensively

| Never | Seldom | Sometimes | Often | Always |
| --- | --- | --- | --- | --- |
| 1.  | 2.  | 3.  | 4.  | 5.  |

7. I have a choice in deciding how I do my work

| Never | Seldom | Sometimes | Often | Always |
| --- | --- | --- | --- | --- |
| 1.  | 2.  | 3.  | 4.  | 5.  |

8. If work gets difficult, my colleagues will help me

| Never | Seldom | Sometimes | Often | Always |
| --- | --- | --- | --- | --- |
| 1.  | 2.  | 3.  | 4.  | 5.  |

9. I am clear about what my duties and responsibilities are

| Never | Seldom | Sometimes | Often | Always |
| --- | --- | --- | --- | --- |
| 1.  | 2.  | 3.  | 4.  | 5.  |

10. Are you currently working in a Magnetic Resonance Imaging (MRI) Unit, or have you worked there in the past 12 months?

Yes 1. **No 2.** **if no, please, go to question number 16**

11. What is the Tesla (T) strength of the MRI machine of your unit? (please indicate only one answer)

*a) only one type of machine in the unit according to T strength*

≤ 1.0 T 1. 

1.5 T 2. 

≥ 3.0 T 3. 

1. *at least two types of machines in the unit according to T strength*

≤ 1.0 T and 1.5 T 4. 

≤ 1.0 T and ≥ 3.0 T 5. 

1.5 T and ≥ 3.0 T 6. 

*c) at least three types of machines in the Unit according to T strength*

≤ 1.0 T, 1.5 T and ≥ 3.0 T 7. 

*In case of work with MRI strengths >3 T (e.g. 4 T, 7 T, 8 T, etc.), please give more details on the strength and on the use (purpose of the use, e.g. for research, diagnosis, or other purposes, daily time of use, year of first use):* ……………………………………………………………………………………………………………………………………………………………………………………

12. Please indicate the year when you started working in an MRI unit and leave blank after *“to”* if you are still working there. In answering, please consider the type(s) of MRI machine(s) according to T strength.

(a) from ………….……… to ……………..…… with ≤ 1 T machine(s)

(b) from ……………….… to ………………..… with ≤ 1.5 T machine(s)

(c) from ……………..…… to ……………….… with ≥ 3 T machine(s)

13. How many hours during your daily work shift, on average, do you work in the MRI unit with the possibility of accessing the “Controlled Access Area” (please base your estimates on the last 12 months).

It is not possible to provide this estimate

It is possible to provide this estimate and the average daily hours are:

≤ 1 hour 1-5 hours ≥ 5 hours No work with this type of MRI

(a) with ≤ 1 T machine(s)    

(b) with 1.5 T machine(s)    

(c) with ≥ 3 T machine(s)    

14. How many MRI procedures, i.e. execution of an MRI examination of a patient, did you attend and/or perform during the last 12 months working in an MRI unit inside the “Controlled Access Area”?

 It is not possible to provide this estimate

 It is possible to provide this estimate and the average annual number of procedures attended and/or performed is: (please indicate the average number of procedures in the blank space if you have worked with the type of MRI machine shown)

 (a)………… with ≤ 1 T machine(s) no work with this type of MRI

 (b)………… with 1.5 T machine(s) no work with this type of MRI

 (c)…………. with ≥ 3 T machine(s) no work with this type of MRI

15. The kind of noise you think is most similar to that of the MRI machinery is:

Fridge 1. 

Hairdryer 2. 

Lawn mower 3. 

Hammer drill 4. 

**Health status**

16. What is your level of agreement or disagreement with the following sentence “Your health conditions are good”:

| Strongly disagree | Disagree | Neutral | Agree | Strongly agree |
| --- | --- | --- | --- | --- |

    

17. In 1936 Hans Selye defined stress as “*the non-specific response of the body to any demand placed upon it”*. When a person cannot face the demands posed by the working and/or private life, stress can become a negative phenomenon (distress) and can cause symptoms such as tension, restlessness, irritability, anxiety or insomnia. Have you experienced this kind of stress in the last 12 months?

| \| Not at all \| Slightly \| Moderately \| Definitely \| Extremely \| \| --- \| --- \| --- \| --- \| --- \| |  |  |  |  |
| --- | --- | --- | --- | --- | --- | --- | --- | --- | --- |

    

18. Do you suffer from car sickness when traveling as a passenger?

| Don’t know | Never | Seldom | Sometimes | Often/Always |
| --- | --- | --- | --- | --- |

    

19. Do you suffer from seasickness?

| Don’t know | Never | Seldom | Sometimes | Often/Always |
| --- | --- | --- | --- | --- |

    

20. Do you experience dizziness or feel faint when you stand up quickly?

| Don’t know | Never | Seldom | Sometimes | Often/Always |
| --- | --- | --- | --- | --- |

    

**If you are not a MRI operator, please answer only the following item 21, then skip to item 32.**

|  | **21. During the last 12 months did you experience any of the following symptoms? How frequently?** | | | **22. Do you feel that the symptom(s) are caused and/or aggravated by your MRI work in the “controlled access area”? If so, please tick the appropriate box for the symptom(s)** | | |  |
| --- | --- | --- | --- | --- | --- | --- | --- |
|  | Never/less than once per month | At least once per month | 1-4 times per week | More than 4 times per week | **Yes** |  | |
| a) Vertigo | 1. | 2. | 3. | 4. | 1. |  | |
| b) Nausea | 1. | 2. | 3. | 4. | 1. |  | |
| c) Concentration problems | 1. | 2. | 3. | 4. | 1. |  | |
| d) Memory loss | 1. | 2. | 3. | 4. | 1. |  | |
| e) Drowsiness | 1. | 2. | 3. | 4. | 1. |  | |
| f) Headache | 1. | 2. | 3. | 4. | 1. |  | |
| g) Metallic taste | 1. | 2. | 3. | 4. | 1. |  | |
| h) Balance instability | 1. | 2. | 3. | 4. | 1. |  | |
| i) Magnetophosphenes | 1. | 2. | 3. | 4. | 1. |  | |
| l) Tinnitus | 1. | 2. | 3. | 4. | 1. |  | |
| m) Sleep disorders | 1. | 2. | 3. | 4. | 1. |  | |
| n) Other symptom(s). Please describe them:  n1)..................................  n2)..................................  n3).................................. | 1.  1.  1. | 2.  2.  2. | 3.  3.  3. | 4.  4.  4. | 1.  1.  1. |  | |

**If you answered “Never/less than once per month” for all the symptoms of item 21, please go directly to item 30.**

23. During the last 12 months did you request a medical examination for any of the above-reported symptoms?

No  Yes 

If yes, a) For which symptom(s)? b) After the medical examination, were you diagnosed with an illness? What kind? c) Did you have to take drugs or other therapies for the symptom(s)? Which drug/therapy?

1. …………………………………………………………………………………………………………..………………………………………………………………………………………………………….
2. …………………………………………………………………………………………………………..…………………………………………………………………………………………………………..
3. …………………………………………………………………………………………………………..…………………………………………………………………………………………………………..

**Please answer the next five questions (24-28) only if you previously answered “yes” to item 22 for any symptom(s) (i.e. you feel that the symptom(s) reported were caused and/or aggravated by your MRI work in the “controlled access area”)**

|  | **24. When do the symptom(s) appear after you start your work shift in the MRI “controlled access area”?** | | | **25. When do the symptom(s) disappear after you finish your work shift in the MRI “controlled access area”?** | | |
| --- | --- | --- | --- | --- | --- | --- |
|  | < 15 minutes | 15-45 minutes | > 45 minutes | < 15 minutes | 15-45 minutes | > 45 minutes |
| a) Vertigo | 1. | 2. | 3. | 1. | 2. | 3. |
| b) Nausea | 1. | 2. | 3. | 1. | 2. | 3. |
| c) Concentration problems | 1. | 2. | 3. | 1. | 2. | 3. |
| d) Memory loss | 1. | 2. | 3. | 1. | 2. | 3. |
| e) Drowsiness | 1. | 2. | 3. | 1. | 2. | 3. |
| f) Headache | 1. | 2. | 3. | 1. | 2. | 3. |
| g) Metallic taste | 1. | 2. | 3. | 1. | 2. | 3. |
| h) Balance instability | 1. | 2. | 3. | 1. | 2. | 3. |
| i) Magnetophosphenes | 1. | 2. | 3. | 1. | 2. | 3. |
| l) Tinnitus | 1. | 2. | 3. | 1. | 2. | 3. |
| m) Sleep disorders | 1. | 2. | 3. | 1. | 2. | 3. |
| n) Other symptom(s). Please describe them:  n1).....................................  n2).....................................  n3)..................................... | 1.  1.  1. | 2.  2.  2. | 3.  3.  3. | 4.  4.  4. | 1.  1.  1. | 1.  1.  1. |

26 a. Have you noticed specific circumstances that you feel are associated with an appearance and/or worsening of the symptom(s) when you are performing your work shift in the MRI “controlled access area”? No 2. Yes 1.

26 b. If yes, please describe these circumstances (e.g. your position or movements with respect to the MRI scanner, the specific task being performed, the type of MRI examination, etc.)

………………………………………..……………….…………………………………….................

……………………..……………………….…………………………………………………………

27 a. Have you taken any drug(s) or other therapies or preventive measures for the reported symptom(s)?

No  Yes 

27 b. If yes, please report details on the drug(s)/therapy/preventive measure(s): ………………………………………..……………….…………………………………….................

……………………..……………………….…………………………………………………………

|  | **28. Thinking about the period when you first started your work as MRI operator, do you remember whether any of the symptom(s) have changed over time (e.g. in intensity or frequency of occurrence)?** | | | | **29. Continuing to work as an MRI operator, have you noticed any change in the frequency and/or intensity of the reported symptom(s)?** | | |
| --- | --- | --- | --- | --- | --- | --- | --- |
|  | I don’t remember | Appearance of a new symptom/ increase of its intensity or frequency of appearance | Decrease of intensity or frequency of appearance of the symptom | Disappearance of the symptom | I have not noticed any change | Decrease of intensity or frequency of appearance of the symptom | Increase of intensity or frequency of appearance of the symptom |
| a) Vertigo | 1. | 2. | 3. | 4. | 1. | 2. | 3. |
| b) Nausea | 1. | 2. | 3. | 4. | 1. | 2. | 3. |
| c) Concentration problems | 1. | 2. | 3. | 4. | 1. | 2. | 3. |
| d) Memory loss | 1. | 2. | 3. | 4. | 1. | 2. | 3. |
| e) Drowsiness | 1. | 2. | 3. | 4. | 1. | 2. | 3. |
| f) Headache | 1. | 2. | 3. | 4. | 1. | 2. | 3. |
| g) Metallic taste | 1. | 2. | 3. | 4. | 1. | 2. | 3. |
| h) Balance instability | 1. | 2. | 3. | 4. | 1. | 2. | 3. |
| i) Magnetophosphenes | 1. | 2. | 3. | 4. | 1. | 2. | 3. |
| l) Tinnitus | 1. | 2. | 3. | 4. | 1. | 2. | 3. |
| m) Sleep disorders | 1. | 2. | 3. | 4. | 1. | 2. | 3. |
| n) Other symptom(s). Please describe them:  n1).....................................  n2).....................................  n3)..................................... | 1.  1.  1. | 2.  2.  2. | 3.  3.  3. | 4.  4.  4. | 1.  1.  1. | 1.  1.  1. | 1.  1.  1. |

30. Do you have an implanted intra-uterine device (IUD)?

| Yes | No | Not applicable |
| --- | --- | --- |
|  |  |  |

31. If yes, have you noticed the appearance of metrorrhagia (i.e. unusual or excessive bleeding during your menstrual period, or intermenstrual bleeding), or changes in the metrorrhagia, after you started your work as an MRI operator?

| Yes | No | Not applicable |
| --- | --- | --- |
|  |  |  |

32. Have you ever been diagnosed with any of the following disease(s)?

|  | Yes | No |
| --- | --- | --- |
| (a) Cerebrovascular disease(s) |  |  |
| (b) Disease(s) of the middle and/or inner ear |  |  |
| (c) Hypertension or hypotension |  |  |

33a. Do you frequently (e.g. on a daily or weekly basis) take any drug(s)?

No 

Yes 

33b. If yes, which drug(s)? ………………………………………..……………….…………………………………….................

……………………..……………………….…………………………………………………………

33c. How often? ………………………………………..……………….…………………………………….................

……………………..……………………….…………………………………………………………

34a. Do you take drug(s) for headache? No  Yes 

………………………………………..……………….…………………………………….................

……………………..……………………….…………………………………………………………

34b. If yes, how often?

………………………………………..……………….…………………………………….................

……………………..……………………….…………………………………………………………

34c. If yes, can you estimate the usual dosage you take?

………………………………………..……………….…………………………………….................

……………………..……………………….…………………………………………………………

35a. Do you feel that any of the following factors are particularly disturbing during your work activity? (you can tick more than one box)

1. Many hours in front of a computer screen 
2. Noise 
3. Inadequate lighting in the workplace 
4. Inadequate temperature in the workplace 
5. Inadequate spaces in the workplace 
6. Manual handling of heavy loads / patients 
7. Other factor(s) 

35b. If you answered “other factor(s)” to 35.a, please describe them:

…….…………………………………………………………………………………………………

…….…………………………………………………………………………………………………

…….…………………………………………………………………………………………………

…….…………………………………………………………………………………………………

36a. Do you have any comments related to the above questions or any other relevant issue to report?

No  Yes 

36b. If yes, please give details:

…….…………………………………………………………………………………………………

…….…………………………………………………………………………………………………

…….…………………………………………………………………………………………………

…….…………………………………………………………………………………………………

…….…………………………………………………………………………………………………

…….…………………………………………………………………………………………………

…….…………………………………………………………………………………………………

…….…………………………………………………………………………………………………

…….…………………………………………………………………………………………………

…….…………………………………………………………………………………………………

…….…………………………………………………………………………………………………

…….…………………………………………………………………………………………………
